# Recent advances in the chemo-biological characterization of decalin natural products and unraveling of the workings of Diels–Alderases

**DOI:** 10.1186/s40694-022-00139-6

**Published:** 2022-04-29

**Authors:** Kenji Watanabe, Michio Sato, Hiroyuki Osada

**Affiliations:** 1 Department of Pharmaceutical Sciences, University of Shizuoka, Shizuoka, 422-8526 Japan; 2 Chemical Resource Development Research Unit, RIKEN Center for Sustainable Resource Science, Wako-shi, 351-0198 Japan

**Keywords:** Diels–Alder reaction, Biosynthesis, Polyketide synthase, Nonribosomal peptide synthetase, Reaction mechanism, Stereoselective reaction

## Abstract

The Diels–Alder (DA) reaction refers to a [4 + 2] cycloaddition reaction that falls under the category of pericyclic reactions. It is a reaction that allows regio- and stereo-selective construction of two carbon–carbon bonds simultaneously in a concerted manner to generate a six-membered ring structure through a six-electron cyclic transition state. The DA reaction is one of the most widely applied reactions in organic synthesis, yet its role in biological systems has been debated intensely over the last four decades. A survey of secondary metabolites produced by microorganisms suggests strongly that many of the compounds possess features that are likely formed through DA reactions, and most of them are considered to be catalyzed by enzymes that are commonly referred to as Diels–Alderases (DAases). In recent years, especially over the past 10 years or so, we have seen an accumulation of a substantial body of work that substantiates the argument that DAases indeed exist and play a critical role in the biosynthesis of complex metabolites. This review will cover the DAases involved in the biosynthesis of decalin moieties, which are found in many of the medicinally important natural products, especially those produced by fungi. In particular, we will focus on a subset of secondary metabolites referred to as pyrrolidine-2-one-bearing decalin compounds and discuss the decalin ring stereochemistry and the biological activities of those compounds. We will also look into the genes and enzymes that drive the biosynthetic construction of those complex natural products, and highlight the recent progress made on the structural and mechanistic understanding of DAases, especially regarding how those enzymes exert stereochemical control over the [4 + 2] cycloaddition reactions they catalyze.

## Introduction

Decalin skeletons have been found frequently among secondary metabolites that are produced by microorganisms such as fungi and actinomycetes. The bicyclic skeleton usually serves as the core structure for various substitutions including an acyl or a pyrrolidinone. As such, decalin-derived compounds exhibit rich structural diversity and exert a wide range of biological activities, such as antibacterial, antiviral, antitumor and antihyperlipidemic activities. Because of the complexity of chemical structures and interesting biological activities of the decalin-based natural products, they have become the target of chemical synthesis and biosynthesis and are being studied all over the world [1, 2].

Based on their biosynthetic origin, decalin ring-containing natural products are broadly classified into two groups: terpenoids and polyketides (PKs). Decalin ring-containing terpenoid compounds mainly belong to sesquiterpenes and diterpenes, and their structures, biological activities and biosynthesis are reviewed in detail elsewhere [3–6]. While the ring formation reactions in terpenoid biosynthesis is considered to proceed predominantly through a carbocation cyclization cascade [5, 6], it has been proposed that the decalin rings found in many PK-derived compounds are constructed by an enzymatic intramolecular Diels–Alder (IMDA) cycloaddition reaction [1, 7]. The existence of enzymes that can catalyze an IMDA reaction, namely Diels–Alderases (DAases), was experimentally indicated as early as the 1990’s [8] and has been debated extensively over the years [9, 10]. Recently, many reports on the discovery of DAases and characterizations of their detailed biosynthetic mechanisms were published in succession [11]. However, no review, including those that are focused on the DAases that catalyze the construction of decalin rings from PK scaffolds, has been reported recently.

In this review, we will start with the discussion of the pioneering works on the biosynthesis of solanapyrone and monacolins as a brief introduction to the history of the discovery and establishment of the existence of DAases. We will then make a comprehensive account of pyrrolidine-2-one-bearing decalin-forming DAases reported with an emphasis on the stereochemistry of the decalin rings and the biological activities of those compounds. A concise review at this point in time is warranted, as the field has recently seen a substantial advancement in terms of the isolation and characterization of this class of natural products as well as identification and biochemical and structural analysis of lipocalin-type DAases that comprise the subset of DAases responsible for the formation of pyrrolidine-2-one-bearing decalin natural products. Lastly, we will outline the enzymes and their coding genes that are involved in the biosynthesis of pyrrolidine-2-one type decalin natural products and discuss the current structural and mechanistic understanding of DAases and how they catalyze the pericyclic reactions in a stereoselective manner. By providing an overview of the recent studies on decalin natural products and DAases associated with their biosyntheses, we aimed to clarify what has been achieved so far and what outstanding questions still need to be addressed to advance and apply this exciting field of study further.

## Decalin ring stereochemistry and its construction

**Fig. 1:**
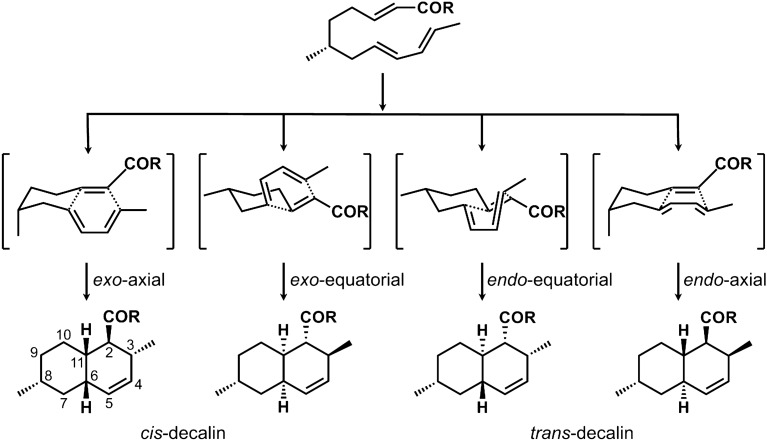
Decalin formation via an intramolecular Diels–Alder reaction, leading to the formation of four stereochemically distinct adducts

The decalin ring biosynthesized from a PK substrate is considered to be constructed through an IMDA reaction involving a triene in the PK backbone. There are four types of cyclization modes a triene system can undergo during an IMDA reaction to form four stereochemically distinct decalin adducts (Fig. 1). While the use of an IMDA reaction of triene on a straight chain is applied frequently as a means of constructing a decalin ring in chemical synthesis, its stereoselectivity is known to depend on various factors, such as the positioning, properties and conformations of the substituents on the substrate, as well as steric, electronic and solvent effects on the transition state. It is also known that the presence of Lewis acid has a great effect on the *cis*–*trans* ratio in the adducts being formed [12–14]. In addition, many studies have been conducted on the stereoselective synthesis of decalin rings, because the stereochemistry of the decalin ring has a great influence on the biological activities of the corresponding compounds [15, 16]. Below, we will describe representative groups of compounds whose biosyntheses have been postulated to involve DAases for their decalin construction.

### Solanapyrones

**Fig. 2:**
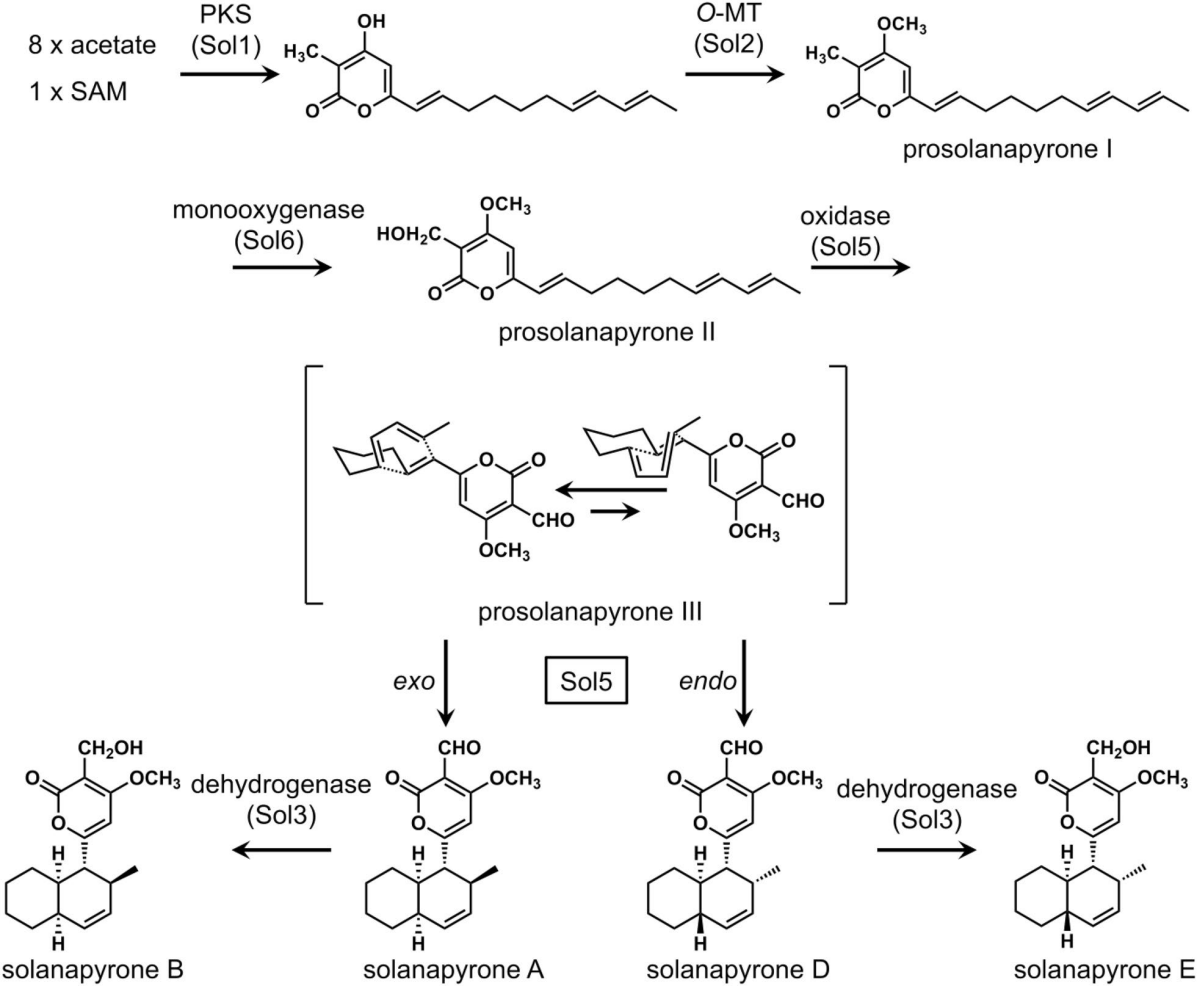
The proposed biosynthetic pathway of solanapyrones, including the oxidation and the cycloaddition steps that are considered to be catalyzed by Sol5 and the four products resulting from the pathway

Natural product solanapyrone is a PK secondary metabolite isolated from the fungus *Alternaria solani* (*Pleosporaceae*) which causes a disease called early blight of potato and tomato [17, 18]. Solanapyrone A (Fig. 2) is a known phytotoxin, and its mode of action was determined to be inhibition of DNA polymerases β and λ [19]. The carbon skeleton of the compound was determined to be of PK origin based on feeding experiments with *A. solani* using labeled precursors [20], and the decalin moiety was thought to be constructed by a biological DA reaction based on the isolation of optically active solanapyrone A reported by Ichihara et al. [21, 22]. To specifically examine the outcome of the cycloaddition step, the linear triene precursors prosolanapyrone I and II labeled with deuterium were synthesized and fed to *A. solani* (Fig. 2) [22]. The result indicated that prosolanapyrone I was oxidized to prosolanapyrone II followed by a [4 + 2] cycloaddition reaction to selectively form solanapyrone A, the expected *exo*-axial adduct of the DA reaction, and B upon reduction of the aldehyde to a hydroxymethyl group (Fig. 2).

In 2010, Fujii et al. isolated the solanapyrone biosynthetic gene cluster (BGC) from the *A. solani* genome and identified the solanapyrone synthase (SPS, Sol5) as a potential gene encoding the solanapyrone DAase [23]. SPS was initially cloned and functionally expressed in the heterologous host *Aspergillus oryzae* to confirm its ability to catalyze the formation of the expected cycloaddition product from the linear precursor. Subsequently, SPS was expressed in large scale in *Pichia pastoris* for a detailed in vitro analysis. The purified enzyme was shown to catalyze an initial oxidation of the achiral prosolanapyrone II to an intermediate prosolanapyrone III before the [4 + 2] cycloaddition reaction takes place to form the *endo* adduct solanapyrone A and the *endo* adduct solanapyrone D in an approximate ratio of 7:1. The results from the study suggested that the primary catalytic function of SPS might be an oxidase activity that converts the hydroxymethyl group in prosolanapyrone II into an aldehyde in prosolanapyrone III that promotes the subsequent DA reaction to form the decalin moiety in solanapyrone A and D. The previous finding indicated that the presence of an aldehyde on the pyrone ring lowers the lowest unoccupied molecular orbital (LUMO) energies of the dienophile [24]. Moreover, the DA reaction was shown to be promoted in aqueous media [24], presumably due to the hydrophobic effect on nonpolar substrates in an aqueous environment [25] and water molecules hydrogen-bonding to the carbonyl groups to activate the dienophile [26]. Taken together, SPS is thought to accelerate the DA reaction by activating the substrate by the oxidation of the substituent, providing a hydrophobic environment that confines the straight-chain triene into a catalytically relevant conformation and furnishing hydrogen-bonding partners to the substrate to further activate the dienophile [23].

### Monacolins

**Fig. 3:**
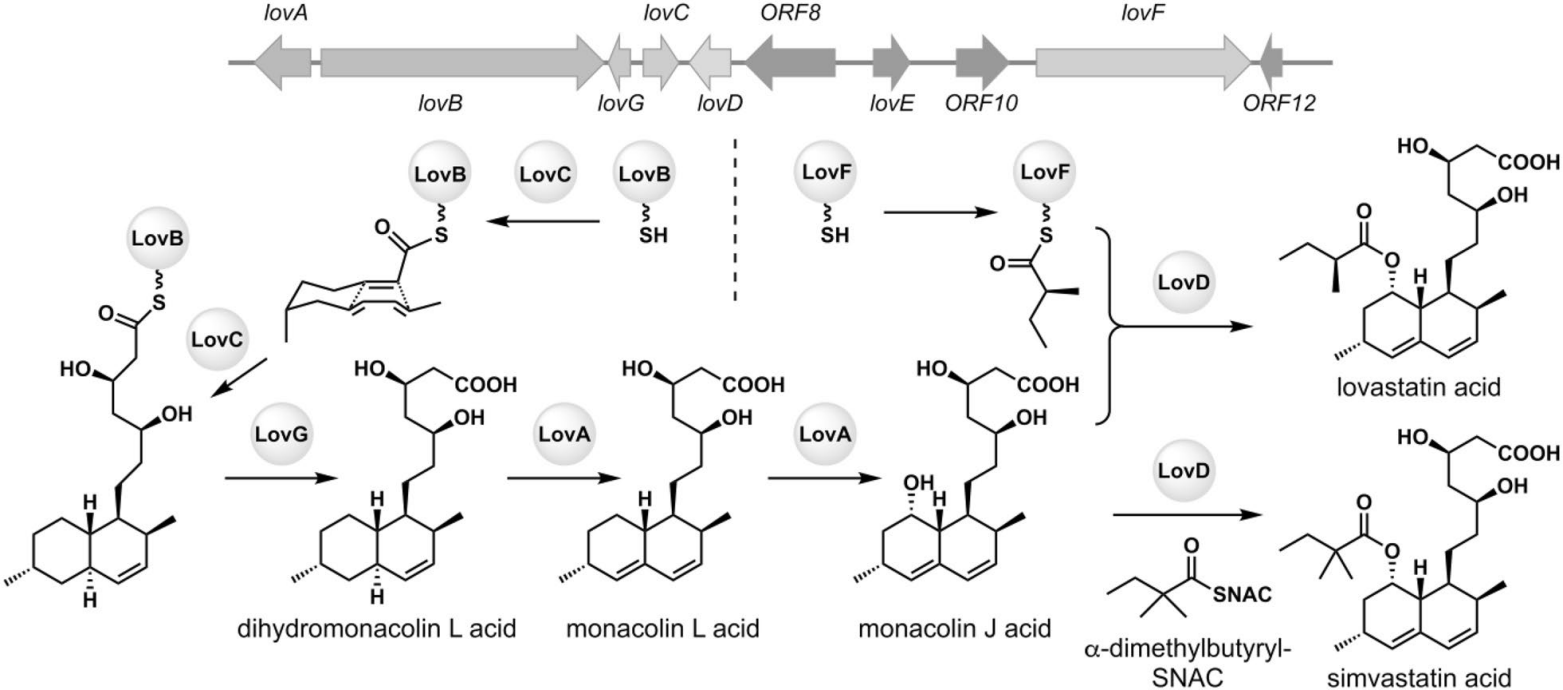
The biosynthetic gene cluster of monacolin and the proposed biosynthetic pathway leading to the formation of the native pathway product lovastatin and the semisynthetic product simvastatin generated upon feeding of α-dimethylbutyryl-*N*-acetylcysteamine thioester (α-dimethylbutyryl-SNAC) [118]

Monacolins are known as PKs of fungal origin that exhibit a significant inhibition of 3-hydroxy-3-methylglutarylcoenzyme A (HMG-CoA) reductase, which catalyzes important steps in the cholesterol biosynthesis [27]. Monacolin K [28], also known as mevinolin and lovastatin [29], is a decalin-containing nonaketide with an acylated C8 hydroxy group, and the semi-synthetic derivative of monacolin J called simvastatin acts as a potent inhibitor of HMG-CoA reductase that is widely prescribed for the treatment of hypercholesterolemia (Fig. 3) [27]. Those monacolins have a rigid and hydrophobic decalin ring core that is linked to a carboxylic acid side chain whose chemical structure resembles the structure of the HMG moiety of HMG-CoA. The crystal structures of HMG-CoA reductase in complex with HMG and simvastatin and other statin-type inhibitors showed that monacolins occupy the binding site of HMG-CoA, thereby inhibiting the original substrate of HMG-CoA to access the active site [27]. The gene cluster for lovastatin biosynthesis was identified in the genome of the producing fungus *A. terreus* [30]. Among the biosynthetic enzymes identified, LovB plays an indispensable role of producing lovastatin. LovB, also known as lovastatin-nonaketide synthase (LNKS), is a highly-reducing iterative polyketide synthase (HR-iPKS) that forms the PK carbon framework of the compound [30]. LovB collaborates with the stand-alone enoyl reductase (ER) LovC to produce a nonaketide intermediate, which remains attached to LovB covalently via a thioester bond (Fig. 3) [31, 32]. In the subsequent studies, in vitro reconstitution of LovB, LovC and the stand-alone thioesterase LovG revealed that the straight-chain polyketide intermediate was transformed to bear a *trans*-decalin ring moiety while attached to LovB, prior to being released by LovG to give the advanced intermediate, dihydromonacolin L [33, 34]. Therefore, unlike the biosynthesis of solanapyrones in which the decalin ring formation is catalyzed by a separate DAase after the PK chain elongation and release by PKS is completed, the lovastatin biosynthesis is considered to accomplish the DA reaction-mediated decalin construction during the process of nonaketide formation catalyzed by the PKS (Fig. 3) [31]. In this respect, LovB also serves as the DAase for the biosynthesis of lovastatin.

### Pyrrolidine-2-one-bearing decalins

**Fig. 4:**
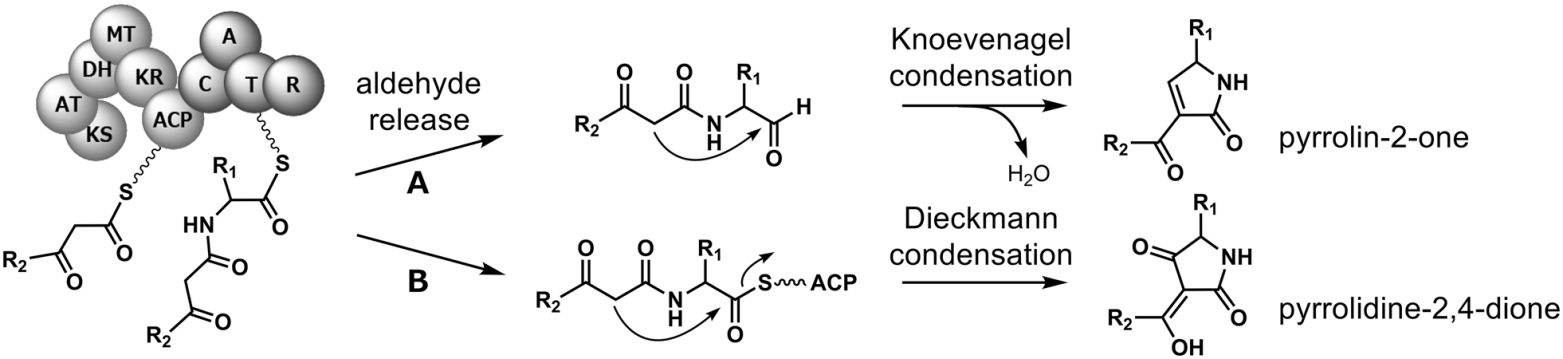
Generalized biosynthetic pathways of pyrrolidine-2-one type natural products catalyzed by a polyketide synthase-nonribosomal synthetase hybrid enzyme. The product can have either a pyrrolin-2one or pyrrolidine-2,4-dione depending on whether the reductase domain-catalyzed cyclorelease reaction proceeds via **A** aldehyde release followed by Knoevenagel condensation or **B** direct Dieckmann condensation. *KS* ketosynthase, *AT* acyltransferase, *DH* dehydratase, *MT* methyltransferase, *KR* ketoreductase, *ACP* acyl carrier protein, *C* condensation, *A* adenylation, *T* thiolation, *R* reduction

The pyrrolidine-2-one-type moieties are known to exhibit important biological activities and commonly found in fungal metabolites biosynthesized through PKS-nonribosomal peptide synthetase (NRPS) hybrid pathways [2, 35–37]. In fact, the lovastatin biosynthetic HR-iPKS LovB carries an inactive domain at its C terminal end that is highly homologous to NRPS-derived condensation domains [30]. This finding, together with the fact that both enzymes are involved in the biosynthesis of decalin-containing products, suggests that LovB might have been derived from a pyrrolidine-2-one-forming PKS–NRPS. In this section, we focus on the pyrrolidine-2-one class of compounds having a decalin ring. Pyrrolidine-2-one derivatives with a decalin ring are composed of two important biosynthetic units. One unit is comprised of a linear carbon fragment of length C14, C16, C18 or longer with several methyl substituents. This linear PK unit can be converted into a decalin ring by an IMDA cycloaddition reaction. The other building block, which is attached to the PK-derived decalin ring, is the amino acid-derived heterocyclic pyrrolidine-2-one moiety that assumes the structure of either a pyrrolin-2-one or a pyrrolidine-2,4-dione. The PK-amino acid product is constructed by a single megaenzyme, a PKS–NRPS hybrid enzyme. Figure 4 illustrates the outline of the biosynthesis of pyrrolidine-2-one class of PK–NRP hybrid compounds by a generic PKS–NRPS enzyme having a typical domain organization. The PK–NRP precursor is released from the PKS–NRPS by the catalytic activity of its terminal reductase (R) domain that yields either pyrrolin-2-one or pyrrolidine-2,4-dione group. The pyrrolin-2-one group is formed via a Knoevenagel condensation after the precursor is released as an aldehyde, whereas the pyrrolidine-2,4-dione group is formed directly by a Dieckmann condensation as the precursor is cleaved from the enzyme (Fig. 4) [38, 39]. Pyrrolidine-2-one-bearing decalin compounds are known to be rich in structural diversity and exhibit various biological activities. In the next section, we will describe the structural diversity and bioactivities of these compounds with an emphasis on the varieties in the pyrrolidine-2-one and decalin moieties and the possible link to the biological activities.

## Diverse structures and biological activities of tetramic acid-bearing decalin natural products

**Fig. 5:**
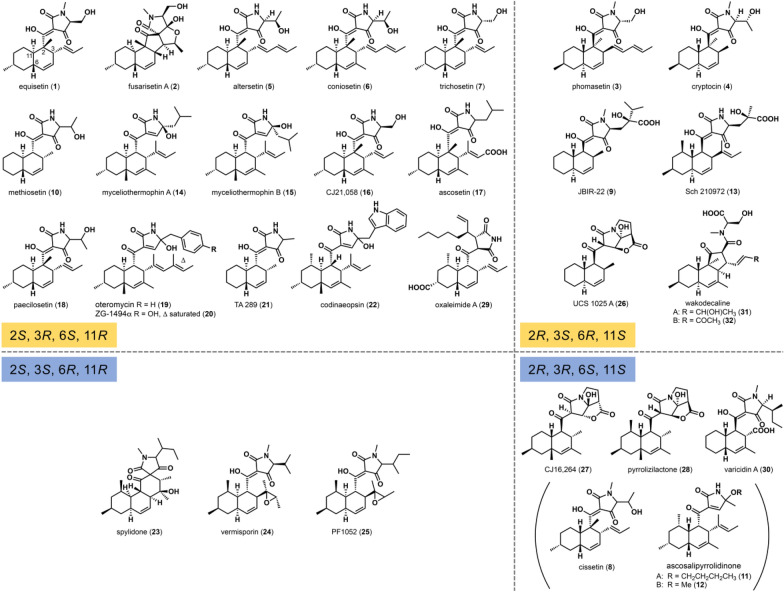
Structural diversity of pyrrolidine-2-one type of compounds categorized according to the stereochemistry at positions 2, 3, 6 and 11 of the decalin moiety that indicates the stereochemical outcome of the decalin-forming Diels–Alder reaction. The top (yellow) and bottom (blue) halves are comprised of *trans*- and *cis*-decalin products, respectively. Compounds within parentheses do not have their absolute configurations determined

Equisetin (**1**) is a representative example of tetramic acid-bearing decalin natural products and known to exhibit a wide range of biological activities against various targets, including broad-spectrum antibacterial activities, cytotoxicity, phytotoxicity and most importantly HIV-1 integrase inhibitory activity (Fig. 5) [36, 40]. This compound was first isolated from *Fusarium equiseti* by Burmeister et al. in 1974 [41, 42]. Compound **1** is characterized by a trisubstituted decalin ring, which also carries a pyrrolidine-2,4-dione moiety derived from a *N*-methylserine residue. The natural product (+)-fusarisetin A (**2**) isolated from *Fusarium* sp. FN080326 was thought to be produced by a similar mechanism with which **1** is biosynthesized based on the similarity in the overall chemical structures of the two compounds [43]. Compound **2** sports a highly unique pentacyclic core structure, and is a potent inhibitor of acinar morphogenesis, cell migration, and invasion in human breast cancer cells [44]. In addition, phomasetin (**3**), an enantiomer of **1** that was isolated from the fungus *Phoma* sp. MF6070 (*Didymellaceae*), exhibited a similar range of IC_50_ values to **1** in a series of assays for the evaluation of their HIV-1 integrase inhibitory activity [45, 46]. To explore the effect of stereochemistry of the compounds on their bioactivity, a pair of epimers of **1** and **3** that differ in the stereochemistry at the alpha carbon of the *N*-methylserine residue were prepared and subjected to the same activity tests. The results indicated that all four compounds exhibited similar HIV-1 integrase inhibitory activities [45].

Cryptocin (**4**) was isolated from *Cryptosporiopsis cf. quercina* (*Dermateaceae*), an endophytic fungus found in the stems of *Tripterygium wilfordii*, a well-known medicinal herb that has been used in traditional Chinese medicine for a long time [47]. Compound **4** resembles **3** in its chemical structure, but interestingly **4** shows a strong activity against certain phytopathogenic fungi, such as *Phytophthora* (water molds that frequently cause root rot in various plants), *Pyricularia* (causative agents of the rice blast disease) and *Fusarium* (causative agents of wilt, blight and basal rot), showing minimal inhibitory concentration (MIC) values in the range of 0.78 to 1.56 µg/mL. On the other hand, it was not active against human pathogenic fungi such as *Candida*, *Aspergillus* and *Histoplasma* [48].

Altersetin (**5**) was isolated as an antibiotic from an endophytic fungus *Alternaria* sp. P 0506 (*Pleosporaceae*) derived from *Vinca minor* (common periwinkle) [49]. The antimicrobial activity of **5** significantly suppressed pathogenic Gram-positive bacteria such as *Streptococcus* and *Enterococcus* species. In particular, **5** exhibited MIC values of 0.5–1.0 µg/mL against various *Staphylococcus aureus* strains in a serial agar dilution assay. However, it did not show any activity against Gram-negative bacteria and pathogenic yeast strains such as *Klebsiella* and *Candida* (Table 1). Interestingly, a modified **5** with its double bonds in the decalin ring and the aliphatic side chain fully reduced showed almost the same biological activity as **5** (Table 1). The biological activity of **1** was also investigated at the same time because of the structural similarity between **5** and **1**. The activity of **1** was similar to that of **5**, and the antimicrobial activity of the reduced form of **1** was also hardly affected (Table 1). These results indicate that the contribution of the decalin and aliphatic side chain toward the biological activity of **1** and **5** is minimal, suggesting that the key motif that furnishes the antimicrobial activity of those compounds might be the tetramic acid moiety.

**Table 1:** Antimicrobial activities of **1**, **5** and their reduced forms against a panel of Gram-positive and negative bacteria

Test organism	MIC [µg/mL]			
	Altersetin (**5**)	Hexahydro- altersetin	Equisetin (**1**)	Tetrahydro- equisetin
*Staphylococcus aureus* 133	0.5	2	1	2
*Staph. epidermidis* 193	1	2	1	2
*Staph. aureus* 44	0.5	2	1	2
*Streptococcus pyogenes* Wacker	2	4	4	8
*Enterococcus faecium* L 4001	4	4	4	8
*Ent. faecalis* 27251	2	2	4	4

Coniosetin (**6**) isolated from the fungus *Coniochaeta ellipsoidea* Udagawa (*Coniochaetaceae*) had a strong growth-inhibiting activity against Gram-positive bacteria, especially multidrug-resistant *S. aureus* at a MIC value of 0.3 µg/mL [50]. The compound closely resembles **5**, where the only difference is that **6** carries an extra methyl substitution on the decalin ring (Fig. 5). Another equisetin-type compound is trichosetin (**7**), which was isolated from the co-culture of the fungicidal fungus *Trichoderma harzianum* with the callus of *Catharanthus roseus* (Madagascar periwinkle) [51]. It could not be isolated from individual cultures of *T. harzianum* or the *Catharanthus roseus* callus. The compound was obtained only when they were co-cultured. Compound **7** is essentially a desmethylequisetin, where the *N*-methylation at the amide group of the tetramic acid moiety of **1** is missing (Fig. 5). Regarding the biological activity of **7**, it showed a strong antimicrobial activity at a MIC value of 1.56 µg/mL against Gram-positive bacteria such as *Staphylococcus* and *Bacillus* strains. However, it did not exhibit any activity against Gram-negative bacteria, yeasts or fungi. Because the pattern of biological activities exhibited by **7** was very close to those exhibited by **1**, the *N*-methylation of the tetramic acid moiety was considered to be non-essential in conferring those compounds with their antimicrobial activities [51].

Among the pyrrolidine-2-one-type compounds identified, cissetin (**8**) isolated from an unidentified fungus OSI 50185 was found to have an unusual *cis*-decalin ring core (Fig. 5). Biological assays were conducted with *cis*-decalin-containing **8** and *trans*-decalin-bearing **1** and **7** to compare their antibiotic activities. However, no significant difference in their antimicrobial activities against Gram-positive bacteria was found. Therefore, the stereochemical conformation of the decalin structure does not appear to play a crucial role in those compounds exhibiting their antibiotic activities. These results suggested that, as the studies on the antibiotic activities of the reduced forms of **1** and **5** also indicated [49], the tetramic acid portion is the key element in conferring to those compounds with their antibiotic activities and the decalin ring is less important in terms of conferring antimicrobial activities to those compounds [52]. Lastly, similar to many of the equisetin-type compounds discussed above, **8** did not show any activity against Gram-negative bacteria as well as yeasts and fungi.

JBIR-22 (**9**) is a natural product produced discovered after successful isolation of a slow-growing rare fungus *Verticillium* sp. F21794 (*Plectosphaerellaceae*) from a soil sample using a screening technique involving the use of daunomycin to suppress growth of ordinary fungi that would grow at a faster rate [53]. Compound **9** was identified as a potent inhibitor of protein–protein interaction involved in the proteasome assembly factor (PAC)1–PAC2 heterodimerization and PAC3 homodimerization that are essential for proper functioning of the 20 S proteasome that is known to sustain high growth rates of cancer cells [54].

Methiosetin (**10**) isolated from a tropical sooty mold *Capnodium* sp. F-190679 (*Capnodiaceae*) found in palm leaf litter from Guatemala was found to possess weak antimicrobial activities at MIC values of 256 and 32 µg/mL against Gram-positive *S. aureus* and Gram-negative *Haemophilus influenzae*, respectively, through a screening method involving the use of antisense to elicit target-based hypersensitivity for identifying antimicrobial compounds and their mechanisms of action [55, 56].

Ascosalipyrrolidinones A (**11**) and B (**12**) represent another example *ci*s-decalin-bearing secondary metabolites (Fig. 5). Those compounds were isolated from the endophytic obligate marine fungus *Ascochyta salicorniae* (*Didymellaceae*) that was isolated from the green alga *Ulva* sp. and carry an unusual deoxytetramic acid moiety. In addition to antimicrobial activities, **11** and **12** exhibited a strong antiparasitic activity against *Trypanosoma cruzi* at a MIC value of 1.1 µg/mL [57].

Sch 210972 (**13**) was isolated from the phytopathogen *Chaetomium globosum* (*Chaetomiaceae*) as a natural product with a strong inhibitory activity against the chemokine receptor CCR-5 at a half-maximal inhibitory concentration (IC_50_) value of 79 nM, exhibiting a potential to be developed into an anti-human immunodeficiency virus type 1 (HIV-1) agent [58]. A series of studies have been conducted on the biosynthesis of this compound to shed light on the mechanism of the DA reaction-mediated decalin formation and the construction of the tetramic acid moiety as discussed in detail below [39, 59].

Five variants of myceliothermophins, another pyrrolidine-2-one-type natural products, were isolated from a thermophilic fungus *Myceliophthora thermophila* (*Chaetomiaceae*). The differences among the group of compounds were concentrated on the leucine residue that makes up the tetramic acid moiety, where myceliothermophins A (**14**) and B (**15**) (Fig. 5) have a hydroxyl group at the α carbon, and myceliothermophins C and D have a methoxy group at the same position. Compound **14** and myceliothermophin C are stereoisomers of **15** and D, respectively. In myceliothermophin E, the leucine α–β bond is unsaturated. Of the five compounds, **14** as well as myceliothermophins C and E exhibited cytotoxic effects on four human cancer cell lines A549, Hep3B, MCF-7 and HepG2. However, **15** and myceliothermophin D did not possess such activities. Thus, once again the configuration of the tetramic acid moiety was implicated to play an important role in conferring interesting biological activities to the decalin-bearing natural products [60].

CJ21,058 (**16**) was isolated from a taxonomically unidentified fungus CL47745 with an antimicrobial activity against Gram-positive bacteria *S. aureus* and *Enterococcus faecalis* with a MIC value of 5 µg/mL. Like many other equisetin-type compounds, **16** was also ineffective against Gram-negative strains such as *Streptococcus pyogenes* and *Escherichia coli*. It was only moderately cytotoxic against HeLa cells with an IC_90_ value of 32 µg/mL. The antimicrobial activity is considered likely due to the inhibitory activity of **16** against SecA, an ATPase that is a member of the multisubunit preprotein translocase complex that controls ATP-dependent transport of secreted proteins across the cell membrane [61].

Ascosetin (**17**) was isolated from an unidentified soil-dwelling ascomycete fungus E-000504855. This pyrrolidine-2-one-type compound carried an unusual carboxylic acid at the beginning of the PK chain and used a leucine residue as the building block for the tetramic acid moiety (Fig. 5). Compound **17** showed antibacterial activities with MIC values of 2–16 µg/mL against various Gram-positive bacteria including *S. aureus* and *Bacillus subtilis*. However, like other equisetin-type compounds, **17** was not active against Gram-negative bacteria except for *H. influenzae*. It was weakly cytotoxic to mammalian cells but not effective against the fungus *Candida albicans* [62].

Paecilosetin (**18**) isolated from the endophytic insect-pathogenic fungus *Paecilomyces farinosus* (*Trichocomaceae*) is another example of a desmethylequisetin with a substitution of the serine residue with a threonine residue that constructs the tetramic acid moiety (Fig. 5). Compound **18** showed an IC_50_ value of 3.1 µg/mL against P388 murine leukemia cells. Unlike vast majority of the pyrrolidine-2-one-type compounds, it also exhibited a growth inhibitory activity against the Gram-positive bacteria *B. subtilis* and the fungi *Cladosporium resinae* and *Trichophyton mentagrophytes* [63].

Oteromycin (**19**) [64], ZG-1494α (**20**) [65] and talaroconvolutins [66] are unique pyrrolidine-2-one-type compounds possessing an extended PK backbone and a deoxytetramic acid moiety derived from either a phenylalanine residue or possibly a tyrosine residue in **20** and talaroconvolutins (Fig. 5). While the molecular formulae of **20** and talaroconvolutin B are identical, the possibility of them being stereoisomers cannot be ruled out due to lack of stereochemical assignments of the key stereocenters of those compounds. Compound **19** was obtained from unidentified fungal species MF 5810 and MR 5811, while **20** and talaroconvolutins A–D were isolated from *Penicillium rubrum* and the ascomycete *Talaromyces convolutus* Udagawa strain NE76-1 (*Trichocomaceae*), respectively. More recently, another group of compounds called cladosporitins A and B were isolated, together with talaroconvolutin A, from the marine fungus *Cladosporium* sp. HNWSW-1 (*Cladosporiaceae*) derived from the mangrove tree *Ceriops tagal*. Cladosporitins also closely resemble **20** except for the occurrence of a succinimide moiety instead of a tetramic acid moiety [67]. Compound **19** was shown to be a novel antagonist of endothelin receptors that may be useful in treating cardiovascular, respiratory, gastric, renal and urological conditions [64], whereas **20** can inhibit the platelet-activating factor acetyltransferase, the enzyme responsible for the last step of the biosynthesis of platelet-activating factor involved in signaling for physiological reactions such as inflammation, allergic responses and anaphylaxis [65]. Interestingly, unlike most other pyrrolidine-2-one-type compounds, **20** and talaroconvolutins B–D were also shown to exhibit antifungal activities [66], and talaroconvolutin A and cladosporitins A and B were cytotoxic against several human cancer cell lines [67].

An unidentified *Fusarium* sp. produces TA-289 (**21**), a very simple type of equisetin-like compounds having only a single methyl group on its decalin ring with the des-*N*-methylated tetramic acid moiety being derived from an alanine residue (Fig. 5). Compound **21** was shown to be toxic to the yeast *Saccharomyces cerevisiae* in a pH- and carbon source-dependent manner and caused irreversible blockage of cell cycle without targeting the microtubules. The study also showed that **21** also caused other biological effects that are less commonly seen among the equisetin-like compounds, such as generation of reactive oxygen species (ROS), increased cell wall permeability and ROS-independent abnormal morphology of mitochondrial [68].

An endophytic fungus CR127A collected from *Vochysia guatemalensis* (white yammer tree), whose internal transcribed spacer (ITS) regions of rDNA was 98% identical in sequence to that of *Codinaeopsis gonytrichoides* (*Chaetosphaeriaceae*), produced codinaeopsin (**22**), an equisetin-like product bearing a previously unreported tetramic acid moiety derived from a tryptophan residue (Fig. 5). Compound **22** showed a growth inhibitory effect against the protozoan human parasite *Plasmodium falciparum* that causes malaria at an IC_50_ value of 4.7 µM [69].

Spylidone (**23**) is unusual in that it not only carries an uncommon *cis*-decalin ring, but also has an unusual tetracyclic core structure with a spiro ring. Compound **23** is produced by a fungus *Phoma* sp. FKI-1840 (*Didymellaceae*) isolated from the soil of Miyakojima Island in Okinawa, Japan [70], along with other previously reported *cis*-decalin compounds vermisporin (**24**) [71] and PF1052 (**25**) [72]. Compound **24** was also isolated from the fungus *Ophiobolus vermisporis* (*Phaeosphaeriaceae*) [73]. Instead of the spiro ring of **23**, **24** and **25** both contain an epoxide on the butyl side chain of the decalin ring (Fig. 5). More recently, simplicilones A and B, a pair of compounds that closely resemble **23**, were isolated from the endophytic fungus *Simplicillium subtropicum* SPC3 (*Cordycipitaceae*) found in the fresh bark of the African medicinal plant *Duguetia staudtii* (Engl. and Diels) Chatrou [74]. While **24** and **25** were found to be antibiotic, **23** did not show any antibiotic activity. Instead, **23** had a biological activity that inhibits mouse macrophages from accumulating lipid droplets [70], which is associated with chronic inflammation that can cause various diseases [75]. On the other hand, vermitrasporin, a possible isomer of **24** isolated from the fungus MSX 105528, was claimed to have an anti-tuberculosis activity [76], and **25** was found to be a neutrophil migration inhibitor [77].

UCS 1025 A (**26**) is a pyrrolizidinone (azabicyclo [3.3.0] octanone) type of natural product (Fig. 5) that is produced together with the hydroxylated analog UCS 1025B by the fungus *Acremonium* (formerly known as *Cephalosporium*) sp. KY4917 (*Hypocreaceae*). Similar to many other pyrrolidine-2-one-bearing decalins described in this review, **26** was active mainly against Gram-positive bacteria, although it was active against a Gram-negative strain *Proteus vulgaris*. Similarly, **26** was only moderately cytotoxic against human cancer cell lines [78]. However, it was shown to have a strong telomerase inhibitory activity [79, 80]. In fact, decalin natural products with the pyrrolizidinone skeleton have been shown to exert a variety of biological activities. CJ16264 (**27**) isolated from CL39457, an unidentified fungus likely an agonomycete strain, was shown to be a broad-spectrum Gram-positive antibiotic with limited activities against Gram-negative bacteria but have a relatively strong cytotoxicity with an IC_90_ value of 8.0 µg/mL [81]. Similarly, pyrrolizilactone (**28**), which was also isolated from an uncharacterized fungus, was determined to be cytotoxic to various cancer cell lines with a proteasome inhibitor activity [82, 83]. It is interesting to note from biosynthetic and bioactivity perspectives that **26** carries a *trans*-decalin moiety while **27** and **28** have a *cis*-configured decalin moiety (Fig. 5).

## New decalin containing compounds isolated by new approaches

In recent years, bioinformatics technology developed rapidly in part due to the advancement of the next-generation sequencing methods that provided vast amounts of genomic information have become available to the scientific community, facilitating the developing new methods and techniques to analyze and exploit the genomic information [84]. Using the so-called Big Data and the continuously advancing array of bioinformatics tools, the new approach of searching for novel secondary metabolites in the genomic information called genome mining has also been practiced actively within the field of natural product biosynthesis in recent years [85]. Such methods have successfully identified several pyrrolidine-2-one-bearing decalin compounds, including oxaleimide A (**29**) and varicidin A (**30**) that were found through bioinformatic mining of the genome sequences of *Penicillium oxalicum* [86] and *P. variabile* [87], respectively (Fig. 5). Compound **29** along with nine other analogs were discovered by identifying a gene cluster in the *P. oxalicum* genome that contained a number of genes, including a DAase-coding gene, known to be involved in the biosynthesis of decalin-bearing secondary metabolites [86]. Compound **30** was obtained by heterologously expressing five key genes from the cryptic BGC, including a predicted DAase gene, found in the genome of *P. variabile*, which was known not to produce any decalin-containing secondary metabolites [87]. Another method that relies on a metabolomic approach to search for new and interesting natural products also led to the identification of novel decalin compounds. Wakodecalines A and B (**31**, **32**) containing a rare cyclopentanone-fused decalin skeleton were isolated from a fungus *Pyrenochaetopsis* sp. RK10-F058 (*Cucurbitariaceae*) along with **3** (Fig. 5) using the Natural Products Plot (NPPlot) method [88]. The NPPlot is a library of semipurified microbial metabolites annoted with UV and LC–MS spectral information that has been used successfully to screen for novel secondary metabolites [89, 90]. Wakodecalines are among many examples of compounds that were identified through such screening activities, and were found to have a moderate antimalarial activity against the *P. falciparum* 3D7 [88]. Wakodecalines might be biosynthesized from **3**, which has the same absolute stereochemistry as wakodecalines, through a spiro-intermediate similar to altercrasin A and its derivatives [91, 92]. As discussed further in “Decalin ring stereochemistry and its effect on the biological activity” section below, it is not difficult to imagine that more decalin-bearing secondary metabolites will be discovered by bioinformatic mining of various fungal genome sequences and metabolomic screening of fungi.

## Decalin ring stereochemistry and its effect on the biological activity

**Fig. 6:**
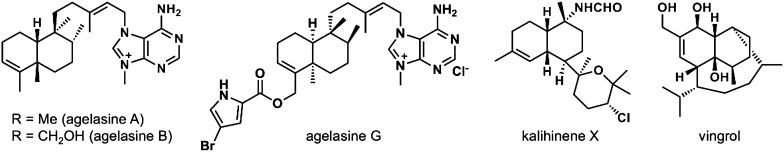
Representative non-pyrrolidine-2-one type natural products containing a *cis*-decalin core structure

Among the currently known fungal secondary metabolites that harbor a decalin ring, the majority falls under the *trans*-decalin type (Fig. 5, top half) and the *cis*-decalin type (Fig. 5, bottom half) is the minority. One of the reasons for the predominance of *trans*-decalin adducts among the natural products is that, while the number of substitutions and the type of functional groups on the linear precursor can greatly affect the outcome of the DA cycloaddition reaction, in general an *endo* cyclization, which leads to the formation of a *trans*-decalin adduct, is kinetically favored to proceed [93]. However, in terms of biological activities of those compounds, *cis*-decalin type of natural products have the tendency to exhibit a wider range of interesting biological activities. For example, the *cis*-decalin-containing diterpene formamide kalihinene X isolated from the marine sponge *Acanthella cavernosa* is known to inhibit attachment and metamorphosis of the cyprid or final mobile form of the larvae of the barnacle *Balanus amphitrite* (Fig. 6) [94]. Similarly, vinigrol isolated from the fungus *Virgaria nigra* F-5408 (*Xylariaceae*) has a *cis*-decaline core with a unique 1,5-butylene bridge [95] (Fig. 6). This compound is a potent antihypertensive agent as well as an inhibitor of epinephrine- or platelet activating factor-induced platelet aggregation with an IC_50_ in the nanomolar range and at the same time an inducer of platelet aggregation with a minimum tolerated concentration in the micromolar range [96, 97]. Interestingly, the *cis*-clerodane diterpene alkaloid agelasine G (Fig. 6) isolated from the marine sponge *Agelus nakamurai* collected in Okinawa, Japan, was found to exhibit antileukemic [98] and protein tyrosine phosphatase 1B inhibition [99] activities. However, agelasine B with a *trans*-decalin core (Fig. 6) but none of the *cis*-decalin-bearing members acts as a Na^+^/K^+^-ATPase inhibitor [100]. Likewise as described in the previous section, *cis*-decalin-bearing **11** and **12** exert a strong anti-trypanosomal activity [57], and **23** acts as an inhibitor of lipid droplet accumulation in macrophages [70]. However, in some cases the stereochemistry of the decalin moiety does not appear to have a significant effect on the biological activities of the compounds as was the case for the antibiotic **8** and its similar *cis* counterparts **1** and **7** [49]. Also, the *cis*-decalin-containing pyrrolizidinone **28** is cytotoxic to various cancer cell lines with a proteasome inhibitor activity [82, 83], but the *trans* relative **26**, while only moderately cytotoxic against human cancer cell lines [78], is a strong telomerase inhibitor [79, 80]. Because there are only a handful of reports of *cis*-decalin type of pyrrolidine-2-one natural products, it is difficult to draw a clear conclusion on the structure–activity relationship on the stereochemistry of the decalin moieties. Nevertheless, due to the structural complexity of these natural products, it is difficult to synthesize and manipulate those compounds synthetically. Thus, there is a growing interest in devising biosynthetic approaches to producing and engineering decalin-containing natural products and their analogs.

## Biosynthetic genes of pyrrolidine-2-one type of decalin ring-containing compounds

**Fig. 7:**
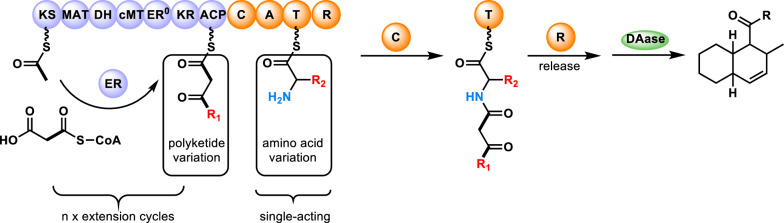
Generalized biosynthetic pathway of pyrrolidine-2-one decalin-containing metabolites produced by the coordinated action of a polyketide synthase (blue)-nonribosomal synthetase (orange) hybrid enzyme, a stand-alone enoyl reductase (ER, blue) and a Diels–Alderase (green)

As briefly discussed earlier, the backbone core of fungal-derived pyrrolidine-2-one-containing natural products is constructed by a PKS–NRPS hybrid enzyme. Song et al. reported the discovery of a biosynthetic enzyme FusA for the production of fusarin C by *Fusarium* fungi, the first hybrid enzyme identified that is responsible for the formation of a pyrrolidine-2-one-containing natural product [101]. To date, biosynthetic pathways of various pyrrolidine-2-one-containing natural products have been examined to uncover the enzymes and the mechanisms involved in their biosyntheses [102]. The biosynthetic process starts from the extension of the PK chain by the PKS portion of the PKS–NPRS hybrid enzyme. In the gene clusters for the biosynthesis of fungal natural products containing a PKS–NRPS gene, there is typically a gene encoding a stand-alone ER that functions in *trans* with the PKS–NRPS [103]. The PKS module and the ER work together to catalyze an iterative elongation of the PK chain. In the meantime, the adenylation (A) domain within the NRPS portion of the hybrid enzyme can activate a preferred free l-amino acid and transfer it to the thiol group at the tip of the phosphopantetheine cofactor covalently linked to the thiolation (T) domain. Once the growing PK chain reaches a pre-set length, the condensation (C) domain of the NRPS module catalyzes the condensation of the PK chain and the thiolation domain-bound amino acid residue to complete the production of the straight-chain precursor PK–NP molecule (Fig. 7). The release of the precursor and the concomitant pyrrolidine-2-one formation catalyzed by the NRPS R domain is as shown in Fig. 4. After the tetramic acid is formed, a [4 + 2] cycloaddition reaction on the triene of the straight polyketide precursor catalyzed by a DAase proceeds to form the decalin moiety. The details of the DAase-catalyzed decalin formation are discussed in the next section.

Biosynthesis of a complex natural product often involves auxiliary enzymes, such as glycosylases, methyltransferases and redox enzymes, that are required to modify the intermediate compounds to generate the final product. Such enzymes are usually encoded together with the genes for the core enzymes, such as PKSs and NRPSs, and regulatory and resistance factors in a single BGC to code for the complete metabolic pathway for the biosynthesis of the target compound. For biosynthesis of pyrrolidine-2-one type of decalin-containing compounds in fungi, generally a PKS–NRPS, an ER and a DAase are encoded in a BGC as a minimally required set of genes. In other words, if a BGC is found to contain genes for a PKS–NRPS, an ER and a DAase, such a BGC can potentially construct a biosynthetic pathway for the production of a decalin ring-containing compound.

Previous studies have shown that such BGCs are widely found in the genomes of various fungal species [104, 105], and variations among the genes encoded in those BCGs contribute to the structural diversity among the pyrrolidine-2-one type of decalin-containing compounds biosynthesized. As such, it is thought that there exist a vast number of decalin ring-containing compounds that are yet to be discovered in nature. In fact, Tang et al. identified the silenced *pvh* gene cluster from the genome of *Penicillium variabile* by using the above set of three genes as query sequence. Upon expressing five genes *pdvA* (PKS–NRPS), *pdvB* (DAase), *pdvC* (ER), *pdvD* (*N*-methyltransferase) and *pdvE* (cytochrome P450) heterologously in *A. nidulans*, the *cis*-decalin-containing compound **30** was successfully obtained [87]. Thus, searching for decalin ring-containing compounds by genome mining is an attractive and effective method for discovering novel metabolites. However, cytochalasan-type of compounds are also considered to be biosynthesized by a PKS–NRPS, an ER and a DAase in a manner that is similar to how decalin-containing compounds are biosynthesized [102]. Therefore, it is still difficult to discern with certainty what secondary metabolite a BGC is programed to produce just from the gene composition of the BGC alone. Furthermore, as was the case for the *pvh* gene cluster discussed above, there are many genetic and epigenetic silencing of secondary metabolite BGCs, especially in fungi. Activation of a silenced BGC through various approaches such as manipulation of transcriptional regulator genes or application of trichostatin A and other small-molecule epigenetic modifiers should facilitate exploration of the untapped potential of fungal biosynthetic capability and identification of various unique biological functions that those secondary metabolites possess [106, 107].

## DAase catalyzing decalin ring formation of pyrrolidine-2-one compounds

**Fig. 8:**
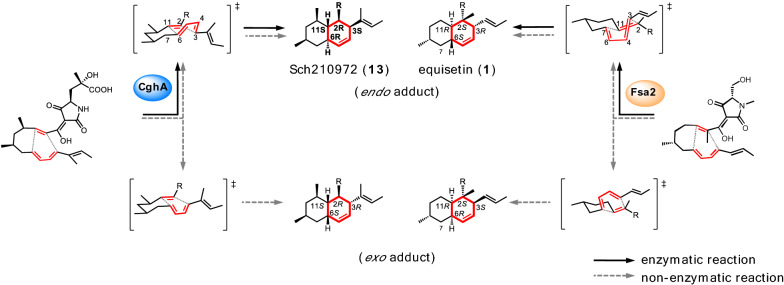
Stereoselective enzymatic Diels–Alder reactions catalyzed by CghA (blue) and Fsa2 (orange), showing the preferential generation of the *endo* adducts by the Diels–Alderases

Regarding the decalin ring-forming DAases involved in the biosynthesis of pyrrolidine-2-one-bearing decalins, Osada et al. reported that Fsa2 present in the BGC of **1** was responsible for the predicted IMDA reaction to form the decalin moiety in **1** [108]. This finding marked the starting point for the research field of DAase-catalyzed decalin biosynthesis. Following the report, Watanabe et al. also showed that CghA, which is also coded in the BGC of **13**, is likely a decalin-forming DAase [59]. Initial amino acid sequence analysis indicated that both Fsa2 and CghA belonged to the lipocalin family of proteins with no clear assignment for their functions. One of the typical functions of lipocalin-type proteins is to bind hydrophobic compounds for transport [109]. Thus, it was speculated that Fsa2 and CghA might also function by binding to the hydrophobic polyketide chains. However, when the genes encoding both of these proteins were deleted from their respective biosynthetic pathways, the formation of unnatural products corresponding to the *exo* adducts of the presumed IMDA reactions was observed in addition to the formation of natural *endo* adducts (**1** and **13**, Fig. 8) [59, 108]. Based on those results, it was strongly implicated that Fsa2 and CghA were the enzymes catalyzing stereoselective DA reactions on linear triene polyketide substrates.

**Fig. 9:**
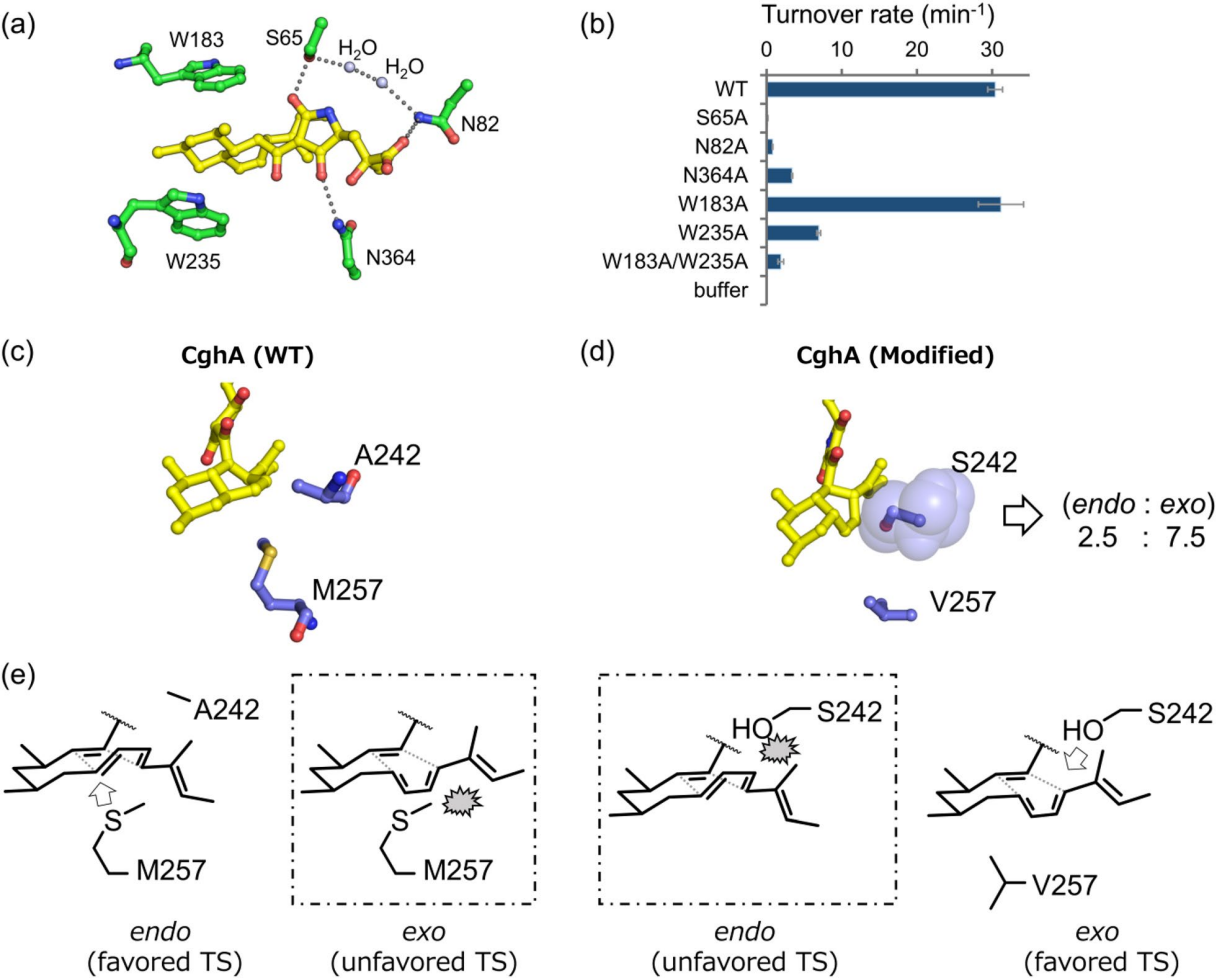
The crystal structure of the CghA–Sch 210,972 complex and engineering of CghA to generate a mutant capable of generating the unnatural *exo* adduct [39]. **a** The structural arrangement of the amino acid residues in the active site of the wild-type (WT) CghA (carbon atoms colored in green) that interact directly with the bound product Sch 210972 (carbon atoms colored in yellow) as observed in the crystal structure of the CghA–Sch 210972 complex. The nitrogen and oxygen atoms are colored in blue and red, respectively. **b** Apparent turnover rates of the conversion of a simplified substrate analog by the WT CghA and its mutants for the formation of the *endo* and *exo* adducts. **c** The amino acid residues (carbon atoms colored in blue) in the active site of the WT CghA in the vicinity of the bound Sch 210972. The sulfur atom is colored in yellow. **d** The amino acid residues (carbon atoms colored in blue) mutagenized within the active site to shift the diastereoselectivity of the Diels–Alder reaction from *endo* to *exo*. **e** Schematic illustrations of the interactions between the side chain groups at residues 242 and 257 and the bound transition state (TS) molecule, indicating the potential steric crashes that can determine the stereoselective outcome of the cycloaddition reaction

While observations of lipocalin-type proteins such as Eqx3 for **1** [110], MycB for **14** [111]. UcsH for **26** [112] and PvhB for **30** [87] that carry out DA reactions for the formation of decalin moieties continued to accumulate, how those enzymes catalyze the cycloaddition reactions remained unclear. However, in a recent study the molecular basis of substrate recognition and catalysis of stereoselective pericyclic cycloaddition reaction by the lipocalinic DAase CghA involved in the biosynthesis of **13** was elucidated [39]. Watanabe et al. co-crystallized the recombinant CghA protein purified from *E. coli* and **13** and determined the enzyme–product complex structure by X-ray crystallography. Based on the structural information, the active site of CghA and the amino acid residues that interact with **13** were clearly delineated (Fig. 9). The large planar sidechain groups from two tryptophan residues Trp183 and Trp235 are coordinated to sandwich the core of the decalin ring, presumably to contribute toward immobilization of the triene polyketide straight chain of the bound substrate within the active site of the enzyme (Fig. 9a). It was also determined that Ser65 and Asn82 formed a water-mediated network of hydrogen bonds with the carbonyl groups of the tetramic acid moiety and the terminal carboxylic acid of the bound ligand. Similarly, Asn364 formed another hydrogen bond with the other carbonyl group of the tetramic acid moiety (Fig. 9a). Since hydrogen-bonding interactions can withdraw electron density and lower the LUMO energy of the dienophile to promote the DA cyclization reaction [26], it was inferred that those residues might also play a catalytic role in accelerating the DA reaction.

To examine these hypotheses on the role of individual active-site residues in catalysis, we prepared CghA with alanine mutation at those residues and analyzed the kinetics of the mutants. Characterization of the mutants confirmed that Trp183 and Trp235 play an important role in catalysis. While the single Trp183Ala mutation did not affect the DAase activity of CghA, the Trp235Ala single mutation reduced the activity by nearly 80%, and the Trp183Ala–Trp235Ala double mutant lost over 94% of the wild-type activity (Fig. 9b) [39]. Similarly, the activities of the Ser65Ala, Asn364Ala and Asn82 mutants were also significantly reduced, suggesting the critical role of hydrogen-bonding interactions at the tetramic acid portion of the molecule in promoting the decalin-forming DA reaction. The loss of activity by the Asn82Ala mutant was particularly interesting, as it indicated that a residue so far removed from the center of reaction could have such a dramatic effect on the catalytic activity of the enzyme. It appeared that Asn82 was important not only for binding the γ-hydroxymethylglutamate side chain to presumably assist in orienting and fixing the substrate within the binding pocket, but also in enhancing the electron-withdrawing ability of Ser65 to accelerate the cycloaddition reaction via the formation of a water-mediate long-range hydrogen-bonding network. The study shed light on the crucial role hydrogen-bonding interactions can play in enhancing the electron attractiveness of the dienophile to accelerate the DA reaction.

The above mutagenesis studies provided some insight into how CghA achieves rate enhancement of the DA reaction. However, they did not address the question of how the enzyme was controlling the stereoselectivity of the cycloaddition reaction. Based on some of the earlier studies of DAase catalytic antibodies [113], it was speculated that the enantioselectivity of CghA was brought about by the conformational constraints placed by the decalin-interfacing amino acid residues on the substrate to facilitate the alkyl chain to preferentially assume the *endo* transition-state structure. Specifically, it was surmised that residues Ala242 and Met257 around the diene moiety of the substrate were particularly important in establishing the shape complementarity of the active site to favor the *endo* conformation over the *exo* conformation (Fig. 9c). Therefore, a Ala242Ser mutation (Fig. 9d) was introduced to increase the steric hindrance against the *endo* transition state (Fig. 9e) while Met257 was shortened to a valine residue (Fig. 9d) to provide the space to accommodate the *exo* transition state within the binding pocket (Fig. 9e). By introducing those two mutations along with an additional Val391Leu mutation, CghA was successfully converted into a DAase that favored the formation of the disfavored *exo* adduct approximately three folds higher than the *endo* adduct [39]. This experiment indicated clearly that active site shape complementarity is key to the stereoselectivity exerted by DAases, and demonstrated the feasibility to engineer the enzyme to favor the formation of naturally disfavored adduct.

**Fig. 10:**
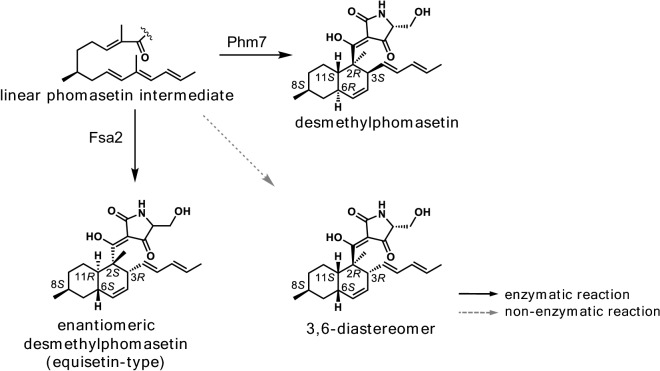
The phomasetin DAase Phm7 catalyzes the [4 + 2] cycloaddition reaction on the phomasetin-type linear triene substrate to generate desmethylphomasetin with a 2*R*,3* S*,6*R*,11* S* decalin moiety, while the equisetin DAase Fsa2 converts the identical substrate into a equisetin-type adduct, whose 2* S*,3*R*,6* S*,11*R* decalin ring is an enantiomer of that found in desmethylphomasetin, demonstrating the opposite stereoselectivity exerted by those two DAases [114]

On the other hand, Kato et al. focused on the biosynthesis of **1** and **3** to uncover the mechanism of stereoselectivity imparted by the lipocalinic DAases on the DA reactions they catalyzed, because the decalin rings of the two compounds are enantiomeric to each other [114]. First, the genome sequence of the fungus *Pyrenochaetopsis* sp. RK10-F058 that produces **3** was searched for a homolog of *fsa2* encoding the DAase that forms **1**, and *phm7* involved in the construction of the decalin ring of **3** was found. Subsequently, by substituting *phm7* with *fsa2* in *Pyrenochaetopsis* sp. RK10-F058, they succeeded in producing an unnatural product that was based on **3** with its decalin stereochemistry following that of **1** (i.e., 2* S*,3*R*,6* S*,11*R*), that is, completely opposite of that of **3** (i.e., 2*R*,3* S*,6*R*,11* S*) (Fig. 10). Thus, the study clearly demonstrated that the enzymes Phm7 and Fsa2 control the stereochemical outcome of the decalin-forming [4 + 2] cycloaddition reaction [114]. The result also suggested high substrate tolerance of the lipocalin-type DAase and opened a new way for producing analogs of pyrrolidine-2-one-bearing decalins having different stereochemistry using molecular genetic techniques. Most recently, the apo crystal structures of Fsa2 and Phm7 were reported [11, 115]. Through extensive computational analyses and docking experiments, a rational explanation as to how the two enzymes catalyze [4 + 2] cycloaddition reactions on the same substrate to generate enantiomerically opposite adducts. Furthermore, the study proposes a comprehensive model for the production of four different diastereomeric decalin scaffolds from the same tetramic acid-bearing linear triene substrate by four types of lipocalinic DAases. The key distinction is thought to be made by how the tetramic acid moiety of the substrate is oriented and the flexible triene polyketide chain is preorganized within the binding pocket of lipocalinic DAases [115]. Those studies will pave the way toward answering the long-standing question about naturally occurring DAases and engineering those fascinating catalysts for biotechnological applications.

## Conclusions

**Fig. 11:**
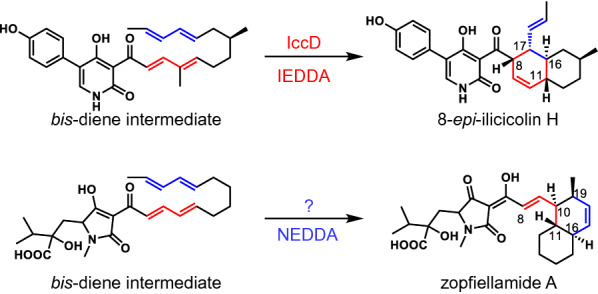
Top row: the DAase IccD catalyzing a decalin core-forming reaction on the *bis*-diene intermediate that proceeds via the inverse-electron demand Diels–Alder (IEDDA) pathway to generate the 4-hydroxy-2-pyridone type compound ilicicolin H [116]. Bottom row: the formation of the decalin core of the natural product zopfiellamide A [117] from its corresponding *bis*-diene intermediate is thought to proceed via the normal-electron demand Diels–Alder (NEDDA) pathway catalyzed by a yet-to-be-identified DAase

In this review, we described the biological activities and biosynthesis of natural products bearing a pyrrolidine-2-one side chain and a PK-derived decalin ring, especially the DAase enzymes that catalyze [4 + 2] cycloaddition reactions for the construction of the decalin ring. This review has focused strictly on lipocalin-type DAases, but there are also other types of DAases that have been discovered to catalyze similar decalin ring constructions. From the recent study on the biosynthesis of ilicicolins, 4-hydroxy-2-pyridone alkaloids, IccD was identified as the enzyme that catalyzes an inverse electron demand Diels–Alder (IEDDA) reaction. Although IccD was annotated initially as a *C*-methyltransferase based on the sequence information, heterologous expression of *iccD* in *A. nidulans* in the presence of a *bis*-diene linear precursor revealed that IccD was in fact a DAase that preferentially catalyzes an IEDDA reaction rather than a normal electron demand Diels–Alder (NEDDA) [116]. On the other hand, as opposed to the IEDDA reaction involved in the biosynthesis of ilicicolins, zopfiellamide type of compounds are generally considered to be biosynthesized through an NEDDA reaction, have also been reported (Fig. 11) [117]. The enzyme responsible for the formation of the decalin moiety of zopfiellamides or the mechanism of how the NEDDA reaction would be catalyzed by a presumed novel DAase have not been uncovered yet.

**Fig. 12:**
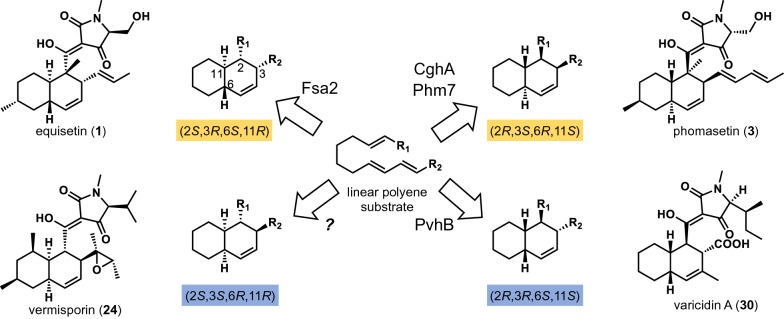
Lipocalin-type DAases can catalyze the generation of four diastereomeric decalin cores of pyrrolidine-2-one-bearing decalin natural products from an achiral linear polyene polyketide substrate [115]. Equisetin-forming Fsa2 represents the 2* S*,3*R*,6* S*,11*R trans-*decalin core-forming DAases. Sch 210,972-forming CghA and phomasetin-forming Phm7 represent the 2*R*,3* S*,6*R*,11* S trans-*decalin core-forming DAases, while varicidin A-forming PvhB represents the 2*R*,3*R*,6* S*,11* S cis*-decalin core-forming DAases. Only the 2* S*,3* S*,6*R*,11*R cis*-decalin core-forming DAase remains to be discovered

To date, lipocalin-type DAases that generate three out of the four possible structural isomers of decalin adducts that can be produced from a linear polyene substrate via a DA reaction have been identified (Fig. 12) [115]. The only remaining type of lipocalin-type DAase is the one that would produce the 2* S*,3* S*,6*R*,11*R* type of decalin-containing natural products represented by compounds such as **24**, **23**, **25** (Figs. 5 and 12). However, it will not be long before the last unknown DAase will be discovered to further deepen our knowledge of the lipocalinic DAases. With the ever-increasing rate of genome sequencing of fungi and other microorganisms, the rapid development of bioinformatics technologies and the advancement of molecular genetics in fungi, we expect speedy discovery of a wider variety of decalin ring-containing compounds and their biosynthetic DAases in the future by making full use of the power of genome mining, genetic manipulation, heterologous biosynthesis and enzyme engineering.

## Data Availability

Not applicable.

## Abbreviations

A: Adenylation
ACP: Acyl carrier protein
AT: Acyltransferase
BGC: Biosynthetic gene cluster
C: Condensation
DA: Diels–Alder
DAase: Diels–Alderase
DH: Dehydratase
ER: Enoyl reductase
HIV-1: Human immunodeficiency virus type 1
HMG-CoA: 3-Hydroxy-3-methylglutarylcoenzyme A
HR-iPKS: Highly-reducing iterative polyketide synthase
IC_50_: Half-maximal inhibitory concentration
IC_90_: 90% maximal inhibitory concentration
IEDDA: Inverse electron demand Diels–Alder
IMDA: Intramolecular Diels–Alder
ITS: Internal transcribed spacer
KR: Ketoreductase
KS: Ketosynthase
LUMO: Lowest unoccupied molecular orbital
LNKS: Lovastatin-nonaketide synthase
MIC: Minimal inhibitory concentration
MT: Methyltransferase
NEDDA: Normal electron demand Diels–Alder
NR: Nonribosomal peptide
NRPS: Nonribosomal peptide synthetase
PK: Polyketide
PKS: Polyketide synthase
PAC: Proteasome assembly factor
R: Reduction
ROS: Reactive oxygen species
T: Thiolation
